# Factors Influencing the Safety Behavior of German Equestrians: Attitudes towards Protective Equipment and Peer Behaviors

**DOI:** 10.3390/ani6020014

**Published:** 2016-02-18

**Authors:** Christina-Maria Ikinger, Jana Baldamus, Achim Spiller

**Affiliations:** Department of Agricultural Economics and Rural Development (DARE), Georg-August University of Göttingen, Platz der Göttinger Sieben 5, 37073 Göttingen, Germany; Jana.Baldamus@t-online.de (J.B.); a.spiller@agr.uni-goettingen.de (A.S.)

**Keywords:** protective behavior, horse, equestrians, horse-related accidents, safety equipment, risk, injury prevention, multiple regression analysis

## Abstract

**Simple Summary:**

The handling and riding of horses can be quite dangerous. Although the use of protective gear among equestrians is increasing, a high number of incidents occur and the voluntary use of safety equipment is described as inconsistent to low. Therefore, this study looks at the safety behavior of German equestrians and at factors influencing this behavior to decrease the high number of horse-related injuries. The results reveal that attitudes towards safety products as well as the protective behavior of other horse owners and riding pupils from the stable are key factors that might alter the safety behavior of equestrians.

**Abstract:**

Human interactions with horses entail certain risks. Although the acceptance and use of protective gear is increasing, a high number of incidents and very low or inconsistent voluntary use of safety equipment are reported. While past studies have examined factors influencing the use of safety gear, they have explored neither their influence on the overall safety behavior, nor their relative influence in relation to each other. The aim of the present study is to fill this gap. We conducted an online survey with 2572 participants. By means of a subsequent multiple regression analysis, we explored 23 different variables in view of their influence on the protective behavior of equestrians. In total, we found 17 variables that exerted a significant influence. The results show that both having positive or negative attitudes towards safety products as well as the protective behavior of other horse owners or riding pupils from the stable have the strongest influence on the safety behavior of German equestrians. We consider such knowledge to be important for both scientists and practitioners, such as producers of protective gear or horse sport associations who might alter safety behavior in such a way that the number of horse-related injuries decreases in the long term.

## 1. Introduction

According to estimates of the Food and Agricultural Organization of the United Nations FAOSTAT for the year 2013, there are approximately 60 million horses worldwide [1]. Equestrianism today encompasses both recreational as well as professional activities that are becoming increasingly popular in many parts of the world [2,3,4]. Human interactions with horses, whether while handling a horse on the ground or mounted, entail certain risks [5,6,7,8]. The literature shows a high rate of horse-related injuries and fatal accidents [6,9,10,11]. Part of the risk is related to the rider’s ability to predict the behavior of the horse. Equestrianism is the only sport that involves a non-human partner that is not only much larger and stronger than its human teammate, but also able to achieve high speeds of up to 65 km/h. Additionally, as a herd and prey animal, the horse has a natural, ethologically predictable flight reflex when facing potentially frightening situations and environments [3,4,5,6,7,12]. To prevent responses from the horse to external frightening stimuli that might endanger the rider, the rider needs to be able to correctly assess and control such flight behavior [13]. Numerous studies stress that horse riding is as dangerous as—or even more hazardous than—other medium- and high-impact sports like motorcycle riding, skiing, automobile racing, football, or rugby [5,7,9,11,14]. Although descriptions of the incidence rate of horse-related injury vary and tend to be underreported, most studies agree that the severity and fatality rate of equestrian injuries can be high [2,10,15]. Differences in risk assessment stem from the unknown number of participants in equestrian sports as well as the unknown number of hours of horse-related activity per equestrian [10]. These numbers are difficult to estimate but reported rates show that one in five equestrians will suffer a severe injury during their riding career [10] and that, compared to other sports, equestrianism has the highest mortality rate with an annual death rate of 1 in 1 million population [11,14,16].

The most common horse-related injuries involve the head, the spine, or the extremities [5,14,17,18,19]. Head injuries are the most serious and most deadly [9,19,20]. In addition to education about horse behavior, safe riding practices, and the proper handling of horses, as well as an increase in personal safety awareness, the use of protective equipment such as helmets or protective vests are commonly recommended strategies to avoid serious horse-related injuries [2,4,7,19,21]. Several studies have already examined and confirmed the effectiveness of helmets within different sports regarding their potential to prevent both frequency and severity of injury, indicating a head injury risk reduction of 60–88%, depending on the type of sport [2,7,22,23,24]. Regarding the equestrian context, previous studies noted a considerable decrease in both the severity and frequency of head injuries due to an increased use and improved design of protective helmets [4,10,18,25]. Although an increasing acceptance and awareness regarding the utility of helmets has already been shown, numerous studies report a high number of incidents and very low or inconsistent voluntary use of safety equipment [4,6,7,14,18,19,26,27]. Most studies regarding helmet use report low rates, with fewer than 40% of riders wearing helmets at the time of injury [3,7,9,12,14]. In contrast to the studies on helmets, there is still a need for research to confirm the effectiveness of protective vests, which are also less commonly used than helmets [2,10,19]. However, the wearing of protective vests is often generally recommended and they are also becoming increasingly popular [2,7].

Some studies have also looked at the reasons behind the refusal to wear protective equipment, in particular regarding equestrian helmets. Multiple psychological, social, and cultural barriers seem to be present [12]. Studies have reported a negative attitude towards helmets among some riders. These equestrians think of helmets as uncomfortable, unnecessary, silly-looking, too expensive, or restricting rider movement [19,26,28]. Some equestrians seem to perceive that horse-riding is not a risky pastime or that the risk is somehow controllable due to experience with and knowledge about horses or familiarity with certain situations and environments [26,27]. Social influences exerted by trainers, family members, peers, and the media may contribute to poor helmet use among riders, too. However, these same influences can also promote the use of protective equipment and act as role models, in particular for young equestrians, simply by adopting the use of protective equipment [26,27]. Furthermore, a certain risk perspective that accepts the unavoidable risk inherent associated with equestrianism regardless of riding experience seems to enhance the use of safety equipment [27]. These results underline the complexity regarding the relationship between risk perception, safety knowledge, attitudes, and protective behavior [27].

A large number of retrospective scientific studies that deal with horse-related risk are now available. These studies have focused on the epidemiology of equestrian-related injuries, identification of risk factors, and the higher-risk groups. They also explore the use and efficacy of technical interventions and safety equipment. In comparison to these studies, research has rarely investigated factors associated with preventing injuries [6,7,19,27,29]. Therefore, examining the preventative measures adopted by equestrians and identifying the influences that promote the adoption of safe practices would be useful [3,6]. Such influencing factors have received comparatively little attention to date. Previous research has tended to focus on the adoption of specific protective equipment (e.g., helmets, vests, boots) or the risk of injury rather than the overall adoption of safety-oriented behavioral practices by equestrians [2,17,26,27]. The present study aims to fill this gap by identifying potential influencing factors and quantifying their impact on the safety behavior of equestrians. An understanding of the drivers for adoption of safety behavior not only informs the scientific discourse but also provides information that can prove useful for producers of equestrian safety equipment or for policy makers regarding, for example, the decision on mandatory helmet use. Administrators of equestrian associations can also use this information to more effectively influence the adoption of safe behaviors by their members. In other fields, such as car driving [30,31], motorcycle riding [32], or bicycle helmet use [33], several studies have already demonstrated that the identification of influencing factors on and determinants of risk behavior provides important information and indications for the design of safety campaigns and for the development of other countermeasures to reduce risk-taking behavior. Identifying influencing factors to alter horse-related safety behavior might therefore also be helpful to reduce the number of horse-related injuries and might further provide useful insights for other high-risk sports and activities where the use of protective gear is recommended, such as cycling, climbing, or skiing.

## 2. Methods

### 2.1. Hypothesis Development

The aim of the present study is to look at potential influencing factors and their impact on the overall safety behavior of equestrians. For this purpose, we measured overall safety as an index including the use of specific protective equipment and the adoption of additional safety measures related to equestrianism (see Section 2.2). In the following, we will briefly present the factors considered here and will derive hypotheses about their potential influence on the protective behavior from the literature.

Several patient surveys repeatedly mention gender as a demographic aspect in connection with horse-related risk. Their results show that the majority of the injured equestrians were young females and conclude that female gender represents a considerable risk factor [2,5,7,17,18,20]. This might be due to the fact that mainly women practice equestrianism [2,7]. However, a multiple regression analysis searching for factors predicting equestrian injury found that the fact that women are more often involved in equestrian activities does not seem to be a reason why women are injured more frequently [17]. Yet, despite the fact that females are getting injured more often, it has also been noted that male equestrians tend to suffer from more serious injuries [4] and less frequently use helmets and other protective gear [26]. In conformity with the more general finding that men are generally less risk-averse than women [34], we assume that:

*H1:*  Female equestrians demonstrate more pronounced safety behavior than their male counterparts.

In view of the link between age and horse-related risk, young riders aged between 10 and 35 constitute the most vulnerable group [4,5,7,18,19]. The share of riding accidents in the total number of fatal sport accidents among children and teenagers is estimated at up to 25% [4]. Some studies found that adolescents take more risks than older individuals, which leads to less pronounced safety behavior [35,36]. In contrast, a study on helmet use within the equestrian community revealed that, due to their lack of experience, young riders perceive themselves as being at a greater risk of injury and tend to wear a helmet more often, while older equestrians are less likely to wear protective gear and, if at all, only do so in certain circumstances that are considered potentially hazardous, such as riding a strange or young and unexperienced horse [26]. Hence, we expect that:

*H2a:*  Older equestrians will show less pronounced safety behavior.

*H2b:*  The age group of children and teenagers protects itself more intensively than older equestrians.

However, perceived social responsibility and the aspiration to act as a behavioral role model positively influence adult behavior regarding safety equipment such as the use of safety helmets. Research has shown this relationship within different kinds of sports and leisure activities, for instance cycling [37], winter sports [38], and also within equestrianism [27]. Scientific research on risk preferences further shows that men and women experience a considerable increase in risk aversion when they have a child, whereas this increase is largest shortly after giving birth and disappears when the child becomes older [39]. Therefore, we assume a positive relationship:

*H3:*  Equestrians with children show more pronounced safety behavior.

Another factor related to horse-related risk is riding experience. Several studies noticed that novice and unexperienced riders constitute a particularly sensitive risk group [4,7,10,15,17,29,40]. In volume terms, a study revealed that the probability for a horse-related injury is three times higher for novice riders compared to moderately experienced riders, five times higher compared to experienced riders, and eight times higher compared to professional riders. In line with this, previous research also found that more years of experience have a decreasing effect on the incidence rate of horse-related injuries [10]. However, several studies agree that experience alone does not necessarily moderate the severity of injuries [4,10] and professional riders’ accidents were found to result in more serious injuries, which may be due to the fact that they train and compete at an increased level of difficulty [7]. Regarding the willingness to wear protective equipment, less experienced equestrians tend to—according to the higher risk they are exposed to—protect themselves more often by means of a safety helmet than experienced riders [10]. Among more skilled equestrians, experience seems to act as a popular argument for foregoing a riding helmet, whereas some even seem to hold the prejudice that wearing protective headgear is automatically associated with being an inexperienced rider and has to be avoided [26,27]. In view of these findings, we expect the following relationships:

*H4a:*  Novice riders protect themselves more than experienced equestrians.

*H4b:*  The more experience a rider has, the less effort s/he puts into protecting himself/herself.

In relation to the opinion that experience can replace other protective measures to prevent serious injury, it can make a difference whether one has already been personally involved in a horse-related accident [27]. Looking at other sports, in the context of alpine ski racers and sky divers some studies have already shown that witnessing the injury of a teammate can lead to fear, which in turn might lead to increased safety behavior [41,42]. We therefore also assume for the equestrian context that:

*H5a:*  The more severe a directly experienced riding accident, the more pronounced the protective behavior.

*H5b:*  The more severe a directly observed riding accident, the more pronounced the protective behavior.

Another risk-related factor is the preferred riding style, as different forms of horse riding can be more dangerous than others due to speed, the need to jump obstacles, or unfamiliar surroundings [15,26]. A study on the most hazardous riding activities revealed that riding outdoors was particularly prone to accidents, followed by dressage, show-jumping, and eventing [19]. Previous research further notes that equestrians practicing one of the English-style riding disciplines (dressage, show-jumping, and eventing (formerly military)) more frequently wear protective helmets [14,26]. Conforming to this finding, helmets are being increasingly employed within a competitive setting [7], with the English-style disciplines traditionally being those with the strongest competitive orientation [43]. For example, in the case of show-jumping, which is also perceived as a higher-risk activity that requires extra protection, riders consider a hard helmet as common [7,17]. Similar thoughts apply to eventing, which represents another equestrian discipline associated with high injury rates [11]. Due to these well-known elevated risks, we expect that equestrians who participate in show-jumping and eventing have a higher acceptance and necessity for safety equipment and we therefore hypothesize that:

*H6a:*  Eventers and show-jumpers show more pronounced protective behavior.

As riding helmets are also known as traditional equipment within dressage riding—except for the rather small group of upper-level dressage riders, who prefer to wear a soft, non-protective hat—and dressage furthermore belongs to the English riding disciplines, which have been observed to have a higher helmet use rate, and, as they are more competition-oriented, are also more likely to accept the use of protective vests [7,14,26]. We expect that:

*H6b:*  Dressage riders show more pronounced protective behavior.

In contrast to the English-style riders, Western riders are less likely to wear a protective helmet because they consider brimmed hats or Stetsons as appropriate headgear within the traditional Western riding culture [7,14,26]. Western riding also belongs to the so-called recreational riding styles, which Havlik [7] found to have much lower helmet use rates and to be less likely to wear protective vests compared to the more competition-oriented English disciplines. We assume that:

*H6c:*  Western riders show less pronounced protective behavior.

As it is still difficult to enforce helmet use in the non-competitive, recreational, or training setting, particularly in riding styles where the classical helmet and the protective vests are not part of the traditionally accepted attire, we further suggest that [7]:

*H6d:*  More recreational riding styles show less pronounced protective behavior.

As already mentioned above, a dangerous but very popular riding activity consists in riding outdoors, which is principally independent of the preferred riding discipline. Horse and rider usually are in more or less unknown surroundings and have less control over their environment, which is one of the reasons why riding outdoors is considered one of the most dangerous pastimes on horseback [6,14,19]. Depending on the surroundings, riding outdoors can also be quite dangerous; in the case of a fall, the ground is likely to be harder than the usually soft riding surfaces, or in the case of the uncontrollable flight of the horse one might hit low branches or even pedestrians or cars. Due to the high risk associated with riding outdoors, we assume that equestrians pay more attention to safety behavior when riding in the countryside compared to riding in familiar environments such as a domestic riding hall or a riding ground and it is postulated:

*H6e:*  Equestrians who spend much of their time with horses outdoors show more pronounced safety behavior.

Several studies also discuss whether there are certain characteristics of the horse, such as breed, type, temperament, or gender, which might represent a risk factor regarding horse-related injuries [6,17,26]. For example, different breeds are associated with certain personalities or specific behaviors such as thoroughbred horses, which rate as rather anxious and excited horses [44] and are less suitable for unexperienced equestrians [4,6]. Research found that typical sport horses such as warmblood horses and thoroughbreds are more reactive [44,45], and hence riding them tends to imply a higher risk. Warmblood horses and thoroughbreds are breeds traditionally used by competition-oriented English-style riders, who show a generally higher helmet and protective vest use rate [7,26]. Therefore, we assume for the overall safety behavior that:

*H7:*  Riders of sport horses such as thoroughbred and warmblood horses show more pronounced safety behavior.

Previous research has already shown the influence of other equestrians on safety behavior like helmet use or other protective measures, e.g., by trainers and other horse owners or riding pupils at the stable [26,27]. This effect is likely to also hold true for the overall protective behavior and therefore we expect that:

*H8:*  The influence of other horse owners or riding pupils from the stable who attach great importance to safety has a positive impact on the protective behavior of equestrians.

Another important aspect regarding equestrian protective behavior relates to the attitude towards specific safety equipment and to horse-related risk perception. For example, previous studies found that negative attitudes towards equestrian helmets—regardless of whether they were perceived as useless, unattractive, or uncomfortable—will exert a negative influence on the willingness to use a protective helmet, whereas a positive attitude can exert a positive influence [26,27]. This finding probably also applies to other items of safety equipment. Accordingly, we assume that:

*H9a:*  A positive attitude towards protective equipment is linked to more pronounced safety behavior.

*H9b:*  A negative attitude towards protective equipment is linked to less pronounced safety behavior.

Finally, in line with findings in the general literature on risk perception that indicate that perceptions of risk can increase preventive behavior [46,47], we address whether the risk perception of the equestrians is influencing protective horse-related behavior [27]. In this context, we expect that the more risky horse-riding is perceived to be by equestrians, the more likely they are to wear protective gear:

*H10a:*  A sensitive risk perception in general leads to more pronounced safety behavior.

*H10b:*  A positive horse-related risk perception leads to more pronounced safety behavior.

### 2.2. Materials and Methods

The data for the present study were collected through an online survey that was open for about six weeks from April to June 2015. The aim of the study was not only to gain information about the protective behavior of equestrians but also to test the effectiveness of a potential campaign for equestrian safety.

The survey comprised four major sections. The first part concerned questions regarding the general horse-related behavior such as riding discipline, experience, skill level, or horse ownership. The second part aimed to collect information about safety-related equestrian behavior such as risk perception and the attitude towards protective gear, the possession and use of safety equipment, former accidents, *etc.* We designed the third part to test the effectiveness of five different safety campaigns and the fourth part comprised questions on general socio-demographic characteristics. We measured most items on a five-point Likert scale. We further utilized single choice, multiple choice, semantic differential, and open questions. The survey used EFS Survey software and was promoted on various German websites and social networks related to equestrian sports such as equestrian journals, horse sport, and breeding associations. In total, 2572 equestrians participated in the survey.

To measure the overall safety behavior, we computed an index based on two factors. The first factor involved the wearing of concrete safety gear such as helmets, safety vests, airbag vests, and combinations of safety and airbag vests in different riding situations (dressage work, jumping/eventing, riding outdoors, riding in the riding hall, riding on the riding arena, and riding unknown horses). We measured the intensity of this behavior on a five-point Likert scale of 1 = Never to 5 = Always. As airbag vests and combinations of safety and airbag vests are worn less frequently (see Section 3.1), we double weighted their score. The second factor concerned additional safety measures such as ensuring that the horse has enough access to a free-range area, that the horse has a balanced and reliable character, the use of safety stirrups, riding outdoors only in a group instead of alone, and lunging the horse before it is ridden. We also weighted these activities according to their prevalence within the sample, with the less frequent activities being more heavily weighted so that high index levels indicated very pronounced safety behavior. The calculated safety behavior index had a theoretical range of 0 to 107 (see Section 3.1).

### 2.3. Analysis

We analyzed the data with the statistics software IBM SPSS Statistics 23. For the purpose of the present study both uni-, bi-, and multivariate procedures were applied. As risk perception and the attitude towards protective equipment were measured by various statements, initially a factor analysis was used for dimensional reduction. In a second step we conducted a multiple regression analysis with the safety behavior index as a dependent variable to identify and determine the impact of significant influence factors.

## 3. Results and Discussion

### 3.1. Sample Description

Of the 2572 respondents, 5.2% were male and 94.8% were female and the average age was 32.5 years (SD: 11.9 years; min.: 12 years; max.: 75 years); 21.4% had one or more children. A large proportion of the respondents had completed their high school education (73.0% had passed the German high school examination). With regard to the basic population of German equestrians, reliable and comparative data is scarce. The only data available originate from the Allensbach Institute for Demoscopy (AWA). Compared to their data from 2014, our sample has a higher-than-average number of females (AWA: 22% male; 78% female) and a higher proportion of respondents who had completed high school education (AWA: 41% with a German high school exam) [48]. However, as comparative data are missing it is difficult to assess whether the AWA figures are also representative. Due to the lack of available data, it is therefore not possible to determine whether our sample is representative for the basic population of equestrians in Germany. Yet, it is a large sample that is suitable for a first exploratory study within the equestrian context.

Related to equestrianism, the participants reported that they had been practicing horse-riding for 19.9 years on average (min.: 1 year, max.: 62 years). Table 1 shows the skill level and preferred riding styles of the participants.

Approximately 98.1% of the participants reported that they had already witnessed a riding accident, with 15.1% ending up in a severe or very severe injury and 68.0% in slight or very slight injury. Another 98.1% reported having been injured themselves, with 15.8% serious and very serious injuries and 64.1% light and very light injuries. The most often affected body parts are shown in Table 2.

The possession and use of safety equipment is shown in Table 3 and Table 4. The calculated safety behavior index was 22.9 on average and varied from 0 to 71.5 (SD: 8.5). The Kolmogorov–Smirnov test on normal distribution was highly significant, rejecting the hypothesis of normal distribution of the index. However, the graphic representation showed a good approximation of normal distribution.

### 3.2. Preliminary Factor Analysis

We conducted an exploratory factor analysis (principal component analysis with orthogonal rotation (varimax)) to reduce the statements regarding the attitude towards safety equipment and risk perception to a lower number of factors. It revealed seven factors that are shown in Table 5.

Both the Kaiser–Meyer–Olkin value (0.887) and the Bartlett’s test of sphericity prove that the present data set is suitable for the application of a factor analysis [49]. We measured the reliability of each factor with the Cronbach’s Alpha value, where values higher than 0.6 indicate a reliable factor, and values higher than 0.5 are accepted in the early stages of research [50,51]. We attained a Cronbach’s Alpha higher than 0.6 for all factors except for factor 7, which only reaches a value of 0.5. However, as its content is quite interesting and the value is not too low, we decided to keep it for further investigation. During reliability analysis regarding factor 6, the statement that aimed at investigating the influence of peer groups “I always wear a helmet/vest, because everybody in our stable does it” did also load on this factor but did not fit well regarding the content of the factor. Elimination of this statement did not decrease the reliability of the factor so we removed it from factor 6.

### 3.3. Multiple Regression Analysis

We computed a multiple regression analysis to test the influence of the discussed factors (see Section 2.1) on the safety behavior of equestrians (for an overview see Table 6). We selected the forced entry method as stepwise regressions are often criticized to be influenced by random variation in the data causing non-replicable results. The model is able to explain 39.2% of the variance of the dependent variable. The ANOVA further confirms that the model, overall, is a significantly good prediction of the safety behavior index as dependent variable [51].

We examined the quality of the multiple regression analysis in terms of multicollinearity, autocorrelation or residuals, and heteroscedasticity. Multicollinearity can represent a serious problem when there is a strong correlation between two or more predictors. We examined both the correlation matrix and the variance inflation index (VIF). The correlation matrix showed only correlations smaller than 0.7 and the VIF was substantially smaller than 10 for all of the predictors. These results show that collinearity does not apply for this model. We looked at the Durbin–Watson statistic to detect the presence of autocorrelation in the residuals. The attained value was very close to 2, showing the assumption of independent errors as fulfilled. We used the plot of standardized residuals against standardized predicted values to test the assumption of homoscedasticity. It revealed that the graph looked slightly like a funnel, indicating moderate heteroscedasticity. To check for cases that might be influencing the regression model, we calculated both Cook’s distance and Mahalanobis distance. Both criteria were fulfilled and less than 5% of cases had standardized residuals above 2 (and 1.6% had standardized residuals above 2.5). In conclusion, there does not seem to be a major problem with influential cases [51].

In total, we included 23 variables in the regression model. Thereof, 17 variables showed a significant influence, while we found no significant influence on the horse-related safety behavior of the variables gender (H1), age (H2a), skill level (H4a), severity of witnessed horse-related accident (H5b), gaited horse riding (H6d), and riding outdoors (H6e). The two variables concerning the general risk perception regarding health risks and high risk and motorsports both exerted a significant influence, yet it was in the opposite direction than expected (H10a). The three variables with the highest influence on the safety behavior proved to be a positive attitude towards safety equipment (Beta = 0.32; *p* < 0.001), followed by the influence of the stable mates (Beta = 0.2; *p* < 0.001), and a negative attitude towards safety equipment (Beta = −0.18; *p* < 0.001).

### 3.4. Discussion

On the basis of previous findings from the literature, we derived 10 hypotheses regarding the influence of various factors on the protective behavior of equestrians, which we subsequently tested by means of a multiple regression analysis. Concerning the influence of gender, we hypothesized that female equestrians demonstrate more pronounced safety behavior compared to male equestrians because they are generally more risk-averse [34] and also tend to be more at risk [2,5,7,17,18,20]. However, we detected no significant effect of gender. Possible causes for this finding may be that horse riding is not classified as hazardous by female equestrians as it is “just” a sport like any other with which they grew up, so they have gotten used to and thus displace the risk inherent to equestrianism. Also, it may be the case that the argument that protective helmets and other safety equipment do not look good plays a more important role for female equestrians and leads to a higher level of rejection [26]. Or it might also be possible that the more risk-averse females do not practice horse-riding at all. If only less risk-averse women choose to practice horse riding, this might be a possible reason why there was no relationship found between gender and safety behavior. It might be that there is no or only a small relationship between gender-specific risk perception and horse-related safety behavior. A significant debate is underway as to whether the high share of women among equestrians may bias the findings that female riders represent a high-risk group for injury [2,7]. In their literature review on horse-related injuries, Hawson *et al.* [4] also found that the younger age groups were predominately female, while the share of men was higher among the older age groups. However, the smaller share of men among the younger age groups was still more likely to get seriously injured. The authors discussed that for certain age groups, especially younger patients, the age- and gender-related distribution of equestrian accidents seems to correspond to that of the basic riding population. Yet this does not seem to hold true for all age groups. For example, the fact that older males are more likely to suffer more serious injury than females in the same age group cannot be explained by the demographic distribution [4]. However, as the share of male equestrians in the sample is quite low (about 5%), the high share of female participants may have somewhat distorted the results, although this is not very likely regarding the large sample size. Looking at the data in more detail, we could find at least some small differences between male and female equestrians. Male riders were older on average, were more often earning their money through riding, and less often rode the same horse but rather different horses. We further found male riders to practice eventing more frequently, while the share of female equestrians riding outdoors was higher compared to their male counterparts. Regarding the use of protective equipment, we found that male participants owned and used a riding helmet less frequently, but were more likely to own an airbag vest. Regarding the perception of horse-related risk, male riders perceived the general riding risks as more dangerous, whereas the female equestrians perceived the special riding risks as being more problematical. This shows that the relationship between gender and protective behavior seems to be highly complex and quite manifold, so that it might be difficult to detect a clear relationship here. It follows that there is a need for further research to clarify the underlying relationships between gender-related risk, risk perception, and safety behavior.

Regarding the influence of age, we hypothesized that younger equestrians in general (H2a) and children and teenagers in particular show more pronounced safety behavior (H2b), but the present results only confirmed the second hypothesis. The finding that young riders tend to be a high-risk group [4,5,7,18,19] and wear helmets more often seems to be reflected in their safety behavior [26]. However, whether this behavior is self-imposed and possibly influenced by a high risk perception on the part of the young riders or rather mandated by parents or trainers remains to be clarified. Also, the requirements for junior riders to wear helmets are more closely mandated by associations, which might also play a role in this context [52]. The finding that older equestrians are less likely to wear protective gear could not be confirmed here [26]. One reason for this could be that the relationship between age and safety behavior is not completely linear. Looking at the correlation between age and the safety behavior index confirms this assumption as we found no significant relationship between them. Although the safety behavior probably decreases with growing years of experience [10,26,27], it might start to decrease—analogous to growing risk aversion when having a child—due to certain events in life such as the birth of a child, increasing domestic or job responsibilities, or getting older in general [39]. A study on attitudes and behaviors towards helmet use revealed similar findings, showing that those were affected by perceived social responsibility and care not only for other riders, but also for relatives, families, and friends [27]. In line with this, the results of the present study confirm that adult equestrians with children show a more pronounced safety behavior compared to those without children, proving Hypothesis 3.

Hypothesis 4a and 4b refer to the relationship between riding experience and safety behavior. Regarding the skill level, the results did not confirm that novice equestrians show a more pronounced safety behavior compared to more advanced or professional riders, although the results did confirm that, in terms of years of experience, the more experienced riders show a less pronounced safety behavior (see Table 6). The finding that experienced riders make less of an effort to protect themselves may have several causes. Firstly, more experienced riders may already have had some probably minor accidents in the past and underestimate the risk for more serious injuries. Also, it could be that as experience increases the control over the horse will improve, which might in turn lead to a false sense of security. In line with the findings by Haigh and Thompson [27], the perception of being able to control the horse and read equine behavior could favor the rejection of safety equipment. As shown by previous research, another possible reason could lie within the fact that some of the more experienced riders seem to relate the use of protective headgear with being an inexperienced rider [26,27] and therefore try to evade the use of protective equipment or other protective measures that might characterize them as newbies. In contrast to more experienced riders, the finding that novice riders do not see the necessity of wearing protective equipment might be influenced by the fact that they might not have experienced or observed a serious fall yet. However, in line with findings indicating that less experienced equestrians tend to—according to the higher risk they are exposed to—protect themselves more often by means of a safety helmet than experienced riders [10], the supposed negative relationship between increasing experience and decreasing protective behavior could again be confirmed. It has to be noted that the classification of riding skills was based on the participants’ self-assessment, which can lead to inaccuracies. Due to the growing heterogeneity within equestrianism, it is increasingly difficult [53], especially across different riding disciplines, to have a reliable skill classification. Hasler *et al.* [17] suggest that a comparable educational level-injury risk index that tracks the true improvement in skills could be a more reliable measure of the relationship between experience and safety behavior. Furthermore, it is possible that not all advanced equestrians reject wearing safety equipment and prejudices might be getting slowly removed. Yet, both novice riders—due to the high number of accidents [4,7,10,15,17,29,40]—and more experienced riders—due to the increased severity of sustained injuries and reduced willingness to exert safety behavior [7,10]—represent rather important target groups for sensitization regarding equestrian-related risks. There is a need for more detailed information regarding the risk awareness and attitudes of these target groups to better assess their behavior. One option to sensitize these target groups would be to use an experienced rider giving a testimonial who serves for both novice as well as advanced riders as an inspiring model giving advice on the safe handling of horses through seminars or courses. Sports celebrities are widely used in classical advertising with the aim of improving awareness and recall, driving sales, and influencing behavior. However, it must be noted that the particular celebrity has to comply with certain conditions and has, for example, to match the product or the topic to be effective [54,55]. More specifically, previous research results have shown that the use of celebrities within public health campaigns can be able to influence health-related attitudes, beliefs and risk behavior and seems to be a promising possibility within the present context [56,57].

Relating to horse accident experience, the high share of each of the 98% of equestrians who stated they had already experienced or witnessed a horse-related accident is consistent with the high number of equestrian injuries found in the literature [9,10,11,14]. The results of the multiple regression analysis confirmed Hypothesis 5a, which assumed a positive influence of the severity of a personally experienced accident on the individual’s protective behavior. However, we found no significant influence for the severity of an observed accident (H5b). It is plausible that accidents that are experienced firsthand arouse a greater awareness of horse-related risks. If the accident has only been experienced indirectly by observation, the personal distance towards the risk seems to be larger and the corresponding risk might be more easily dismissed. Yet this differs from O’Neil’s [41] findings that the injury of alpine ski racers did have a psychological impact on the respective teammates. One reason for this could be that individuals might think they are safe and believe that misfortunes only ever happen to others. However, witnessing traumatic injuries can distort such beliefs. To cope with such traumatic events, people engage in different coping strategies [58]. One of these strategies might consist of mental distancing from the injury. In line with this, a study on helmet use showed that it is being frequently argued by equestrians that one could do without safety equipment as the risk is perceived to be controllable [27]. Such a false belief can be more easily maintained in the case of a witnessed accident as the riders themselves have not lost control and can talk themselves into believing that they might have reacted differently. The extent to which a rider can maintain this false belief might further depend on the observed severity of the injury. However, this is only speculation and it would be interesting to explore whether there is a certain degree of injury necessary to realize a change in safety behavior and what additional factors might play a role regarding the influence of observed accidents. As research in this field is said to focus too heavily on the effects on the direct victim and has frequently neglected the potential effects of serious injuries on the witness, it would be interesting to take a deeper look at this phenomenon within equestrianism and other kinds of sports [58].

Previous research has also found that equestrians who practice one of the more competitive-oriented English-style riding disciplines wear helmets more frequently [7,14,26,43]. The present results confirmed that show-jumpers (H6a), eventers (H6a), and dressage riders (H6b) show more pronounced protective behavior. Also, other studies have showed that the less frequent use of helmets, which is characteristic of Western riding [7,14,26] and other more leisure-oriented disciplines, has [7]—as proposed in hypothesis 6c—a negative effect on the overall protective behavior. In contrast to the riding of gaited horses, which also belongs to the more leisure-oriented riding disciplines, we found no significant relationship. The present results could only partially confirm Hypothesis 6d, which assumes that more leisure-oriented disciplines put less effort in safety behavior. Regarding the relationship between protective behavior and riding outdoors, which several studies consider as one of the most dangerous pastimes on horseback and which often includes a high share of leisure-oriented equestrians of different disciplines, again no significant relationship could be found (H6d) [6,14,19]. It is becoming clear that there are huge variations between the several leisure-oriented riding disciplines and their respective safety behavior. Such information would be helpful for horse sport associations to identify and communicate with more vulnerable groups that have a greater need for safety education. Western riders represent such a vulnerable group, as they often refuse to wear protective helmets as these are not considered appropriate Western-style equipment. In recent years, some producers have tried to develop Western-style protective helmets, but they were not successful on the market. Whether this was due to the look of the helmet, its wearing comfort, or other reasons remains unknown. For the Western riding associations, this implies that it is necessary to work on the development of protective gear that is better accepted by the Western riding culture as well as education about horse-related risk and the advantages and effectiveness of protective gear.

Hypothesis 7 assumed a significant relationship with the breed of the horse. As expected, we could observe a positive relationship between the riding of sport horses such as warmblood or thoroughbred horses and more pronounced safety behavior. It is unclear whether the observed positive relationship between riding of sport horses such as warmblood or thoroughbred horses and more pronounced safety behavior is due to a perception that these breeds are associated with a greater degree of unpredictable behavior and higher risk [44,45].

In line with the findings from the literature, the present results indicate that the influence of social groups and peer groups can positively influence helmet use [26,27]. Yet, the present study only looked at the influence of one social group, namely other horse owners and riding students from the stable. To keep the number of influencing factors manageable, we looked first at the more general group of other horse owners and riding pupils from the stable. That specific group is likely to have a close and horse-relevant contact with an equestrian and hence also the possibility to exert a strong influence on the latter. In view of the strength of the respective impact, this variable constitutes a quite important influence factor, as it exerts the second highest influence overall and provides a valuable starting point for the promotion of safety behavior. Here, especially trainers, stable managers, and horse sport associations are asked to inform their pupils, members, and clients about safety aspects concerning equestrianism, to reduce safety-related prejudices and to establish a positive security culture among the riders in a stable. As we did not further differentiate the group of horse owners and riding pupils from the stable, it would be useful to ask which individuals and subgroups, such as trainers or equestrian idols and also non-riding friends and family members, influence safety behavior at all and which of these groups exert a particularly high impact. Such knowledge could provide further starting points to enhance preventive behavior.

From all the factors examined within the multiple regression analysis, we identified the attitude towards protective gear as the most influential factor. As expected, a positive attitude exercised a positive influence (H9a) and a negative attitude exercised a negative influence (H9b) on protective behavior. The positive attitude exerted the highest impact overall and the negative attitude had the third-largest influence. The attitude towards safety equipment seems to represent a key aspect when trying to increase the use of safety products. Producers of safety equipment and horse sport associations should continue to try to find out more about the underlying reasons for these attitudes to identify potential enablers and barriers. Deficient design, untraditional appearance, lack of comfort, and doubts about the effectiveness of safety products seem to be major reasons for rejection. Therefore, the research into this area must be continued, especially on safety vests as their effectiveness to reduce horse-related injuries has still to be confirmed and has already been questioned within other sports [2]. Furthermore, the design and comfort of safety equipment might be other important aspects to look at. In the case of helmets, the design can contribute decisively to the decision to wear safety equipment [26]. Potentially, if the producers of safety equipment would work on the comfort and look of the product, it might turn into a rather desirable fashion item that riders would more often voluntarily use.

Finally, the last hypothesis expected both a positive influence of risk perception in general (H10a) and horse-related risk perception in particular (H10b) on protective behavior. However, the present results only confirmed the second hypothesis. Surprisingly, we detected a small but significant negative relationship. We measured risk perception in general as the personal risk perception of basic and high risk situations. The basic situation comprised moderate health risks such as having too much stress or insufficient sleep or exercise. The high-risk situations included activities such as extreme sports and motor sports. The reasons for the observed negative relationship with protective behavior are not clear so far and a more detailed examination of the connection between risk perception in general and horse-related risk perception in particular is necessary. Perhaps the phenomenon of risk suppression is a possible explanation. In this sense, more risk-averse people in general, especially when they have finally decided to participate in a high-risk sport or hobby such as equestrianism, might willingly suppress the associated risk, which might be considered quite harmless compared to other high-risk sports. Certain findings in the recent literature partially confirm this phenomenon [26,27], which shows that equestrians generally state that they believe they can control horse-related risk. Future research projects need to scrutinize this assumption. Perception of the level of danger associated with the equestrian activity in general and specific riding situations positively influenced the protective behavior patterns reported by equestrians. This relationship engenders a responsibility of horse sport associations to educate their members about the inherent horse-related risks to produce more safety-oriented behavior in equestrians.

### 3.5. Limitations

As already discussed in the sample description (see Section 3.1), it is unclear whether the sample is representative so the generalizability of the present study may be limited. The present study is subject to self-selection bias in that certain types of respondents participated in the survey. Those with a high interest in horse-related safety may be overrepresented. Therefore, the extent to which the transfer of the results of the present study to the German equestrian population may be limited. However, given the high number of participants and the finding that sociodemographic variables did not exert a strong influence, it is likely that these results provide an important first approximation in this area of study.

The study’s methodological limitations include selected method of analysis and the calculation of the index to measure safety behavior. Although multiple regression analysis is a method able to identify the relationship among two or more variables, this does not automatically imply that this relationship is also causal [59]. However, based on logical considerations, this affects only a small number of variables, such as show-jumping. For instance, it could be that some equestrians might only dare to jump at all because they practice very pronounced safety behavior. Such a relationship could further be linked to the phenomenon of risk compensation, in connection with which it is being discussed whether the wearing of protective gear can also exert the opposite effect on safety behavior, such as the use of protective equipment like helmets or safety vests, giving a false sense of security and promoting risk-prone behavior instead of reducing it [2,7,60]. Another methodological limitation concerns the dependent variable. The safety behavior index mainly covered the use of protective equipment in different riding situations, which might imply that factors concerning protective gear exert a disproportionally high impact. Since several studies showed that not only horse riding itself is dangerous, but also simply handling the horse can lead to serious injuries, such as trampling, being kicked, or being bitten [5,8], it would be interesting to look at the specific protective behavior when handling a horse and compare it to the behavior when riding.

Moreover, it has to be noted that it is difficult to judge the quality of protective gear as it might be useful in the case of serious injury but might not avoid dangerous situations overall [4,14]. It may even be possible, as already discussed above, that some kind of risk compensation is at work such that the wearing of protective gear can also result in riskier behavior [2,7,60]. Future research regarding the use and effectiveness of protective gear but also the impact of additional measures that can reduce horse-related risk could provide additional useful information. As already proposed in the literature, an important additional safety measure constitutes the improvement of the predictability of horses through better education and understanding of equine learning and behavior patterns, building on recent findings from research on horse ethology and equitation science, as it is a commonly cited cause of human injury [4]. In this context, improving riders’ competence in physical skills such as fitness, balance, the proper application of aids, and falling techniques should make them more resilient to injury and falls; establishing clear rules and legislation requiring the mandatory wearing of approved safety gear and increasing general awareness of horse-related risk for both individuals and the general public are further possible measures to reduce horse-related risks. The establishment of good practices and a comprehensive safety management within stables will ensure a safe environment [6,8,12,19].

## 4. Conclusions

The present comprehensive study examined the potential influence and impact of 10 different factors on the safety behavior of equestrians by means of a multiple regression analysis. It should be noted that the relationships between the respective variables are quite complex. The results show that the attitudes towards safety products as well as the protective behavior of other horse owners and riding pupils from the stable are key aspects in altering the safety behavior of equestrians. The obtained outcomes could help horse sporting associations, politicians, and producers of horse-related safety gear find additional starting points for the promotion of risk preventive behavior and identify important high-risk groups that should be made more aware of the various advantages of protective gear. The findings herein may also inform other high-risk sports administrators seeking promotion of more pronounced safety behavior in their participants.

## Figures and Tables

**Table 1:** Skill level and riding styles.

**Skill Level**	**%**
Beginner	30.2
Intermediate	49.0
Advanced	20.8
**Riding Styles *(Multiple Answers Were Possible)***	**%**
Outdoors	82.7
Dressage	75.0
Show jumping	38.9
Eventing (former military)	13.9
Western riding	12.6
Riding of gaited horses	11.5

**Table 2:** Most often affected body parts.

Affected Body Parts	%
Upper extremities	28.8
Lower extremities	15.0
Pelvis	13.0
Spine	10.1
Head	8.1

**Table 3:** Possession of safety gear and other safety measures.

**Safety Equipment (*n* = 2572)**	**%**
Protective helmet	97.7
Safety stirrups	46.7
Protective vest	44.1
Airbag vest	3.3
Combination of protective and airbag vest	0.9
None	2.0
**Other Safety Measures (*n* = 2570)**	**%**
Horse has enough access to a free-range area	84.9
Select horses that show a more predictable behavior	57.7
Riding outdoors only in a group	32.1
Lunging the horse before it is being ridden	10.8

**Table 4:** Percentage of riders always or often using safety equipment in different riding situations (top-2-box).

Riding Situation	Safety Equipment			
	*Protective Helmet*	*Safety Vest*	*Airbag Vest*	*Combination of Safety/Airbag Vest*
Jumping/eventing	96.0 % (*n* = 1911)	78.1 % (*n* = 917)	88.7 % (*n* = 62)	81.0 % (*n* = 21)
Riding of unknown horses	95.6 % (*n* = 2312)	43.8% (*n* = 934)	49.2 % (*n* = 65)	45.0 % (*n* = 20)
Riding outdoors	92.4 % (*n* = 2500)	40.4 % (*n* = 1087)	55.1 % (*n* = 78)	33.3 % (*n* = 21)
On the riding arena	83.0 % (*n* = 2487)	17.2 % (*n* = 1054)	32.4 % (*n* = 74)	9.1 % (*n* = 22)
Dressage work	82.7 % (*n* = 2406)	14.7 % (*n* = 1023)	31.1 % (*n* = 74)	4.8 % (*n* = 21)
In the indoor riding hall	82.1 % (*n* = 2385)	15.1 % (*n* = 1022)	29.2 % (*n* = 72)	9.1 % (*n* = 22)

Note: items were measured on a scale of 1 (always) to 5 (never); the use of the respective safety product is illustrated here as top-2-box including 1 (always) and 2 (often).

**Table 5:** Results of the exploratory factor analysis.

Factor	Variable/Statement	Factor Loading
Factor 1: Perception of general riding risks (Cronbach’s Alpha = 0.869)	Grooming the horse is particularly dangerous.	0.845
	Doing ground work with the horse is particularly dangerous.	0.790
	Riding in the riding hall is particularly dangerous.	0.738
	Grooming the hind legs of a horse is particularly dangerous.	0.732
	Riding in the riding arena is particularly dangerous.	0.721
	The loading of horses is particularly dangerous.	0.531
Factor 2: Negative attitude towards safety equipment (Cronbach’s Alpha = 0.740)	Safety equipment such as helmets or vests is simply uncomfortable.	0.731
	Safety equipment such as helmets or vests look unflattering.	0.725
	Safety products are too expensive; I prefer spending the money on my horse.	0.576
	I want to relax when practicing my hobby and don’t want to think about risks.	0.574
	I believe that safety products do not really protect in the most serious cases.	0.541
	Nothing has ever happened to me when riding; I think the risk is often exaggerated.	0.538
	Safety equipment is just for kids.	0.516
Factor 3: Perception of special riding risks (Cronbach’s Alpha = 0.736)	Jumping in the country is particularly dangerous.	0.774
	Jumping over an obstacle is particularly dangerous.	0.689
	Riding on/beside a country road is particularly dangerous.	0.556
	A prize-giving ceremony during an equestrian event is particularly dangerous.	0.505
	Carriage rides are particularly dangerous.	0.411
Factor 4: Perception of general health risks (Cronbach’s Alpha = 0.717)	Insufficient exercise is particularly dangerous.	0.708
	Insufficient sleep is particularly dangerous.	0.701
	Stress is particularly dangerous.	0.679
	Eating lots of fat and sugar is particularly dangerous.	0.644
	Being exposed to the sun unprotected for a long time is particularly dangerous.	0.517
	Alcohol consumption is particularly dangerous.	0.450
Factor 5: Perception of extreme and motor sport risks (Cronbach’s Alpha = 0.602)	Motorbike riding is particularly dangerous.	0.735
	Extreme sports like sky diving and cliff climbing are particularly dangerous.	0.704
	Fast driving is particularly dangerous.	0.569
Factor 6: Positive attitude towards safety equipment (Cronbach’s Alpha = 0.635)	Riding without helmet is particularly dangerous.	0.597
	Riding without a safety/airbag vest is particularly dangerous.	0.511
	Bicycle riding without a helmet is particularly dangerous.	0.466
Factor 7: Risk averse perception and behavior (Cronbach’s Alpha = 0.501)	I’m willing to spend money on safety equipment.	0.752
	I’m fully aware of the risk of horse riding.	0.708

Note: KMO: 0.887, Bartlett’s test of sphericity highly significant (*p* < 0.001), explained variance: 51.91%.

**Table 6:** Results of the multiple regression analysis.

Variables	Unstand. Coefficients		Stand. Coefficients	Sig.	VIF	Hypothesis
	B	Std. Error	Beta			
(Constant)	18.38	1.37		0.000		
Gender	−0.33	0.71	−0.01	0.650	1.17	H1 not confirmed
Age	0.03	0.02	0.04	0.186	3.07	H2a not confirmed
Children/teenager *vs.* adults	−1.279	0.48	−0.05	0.007	1.41	H2b confirmed
Having children	0.44	0.21	0.04	0.033	1.41	H3 confirmed
Beginners *vs.* intermediate/advanced riders	−0.51	0.34	−0.03	0.133	1.18	H4a not confirmed
Riding experience	−0.05	0.02	−0.06	0.016	2.18	H4b confirmed
Severity of own accident	0.40	0.13	0.05	0.003	1.11	H5a confirmed
Severity of witnessed accident	0.18	0.13	0.03	0.154	1.09	H5b not confirmed
Show-jumping	1.45	0.35	0.08	0.000	1.43	H6a confirmed
Eventing (former military)	2.93	0.44	0.12	0.000	1.19	H6a confirmed
Dressage	0.81	0.40	0.04	0.039	1.41	H6b confirmed
Western riding	−1.51	0.48	−0.06	0.002	1.27	H6c confirmed
Gaited horse riding	0.25	0.50	0.01	0.613	1.28	H6d not confirmed
Riding outdoors	0.40	0.39	0.02	0.307	1.07	H6e not confirmed
Breed (sport horse)	0.96	0.34	0.06	0.005	1.44	H7 confirmed
I always wear a helmet/vest, because everybody in our stable does it.	1.31	0.13	0.20	0.000	1.28	H8 confirmed
Positive attitude towards safety equipment (Factor 6)	2.76	0.19	0.32	0.000	1.84	H9a confirmed
Negative attitude towards safety equipment (Factor 2)	−1.55	0.15	−0.18	0.000	1.15	H9b confirmed
Perception of general health risks (Factor 4)	−0.31	0.15	−0.04	0.040	1.13	H10a not confirmed
Perception of extreme and motor sport risks (Factor 5)	−0.56	0.16	−0.07	0.001	1.28	H10a not confirmed
Perception of general riding risks (Factor 1)	0.38	0.15	0.05	0.013	1.17	H10b confirmed
Perception of special riding risks (Factor 3)	0.53	0.15	0.06	0.000	1.07	H10b confirmed
Risk averse perception and behavior (Factor 7)	0.85	0.15	0.10	0.000	1.08	H10b confirmed

Note: Method = forced entry, *R*^2^ = 39.2%, ANOVA = 0.000; Durbin-Watson: 2.06; Cook’s distance: 0.00; Mahalanobis distance: 22.99; dependent variable = safety behavior index.
